# Retraction: *SOX15* regulates proliferation and migration of endometrial cancer cells

**DOI:** 10.1042/BSR-20171045_RET

**Published:** 2021-04-16

**Keywords:** endometrial cancer, migration, proliferation, SOX15

This article is being retracted from *Bioscience Reports* at the request of the Editor-in-Chief and the Editorial Board following receipt of a notification from a reader, alerting the Editorial Board to three pairs of duplicated images in Figure 3A.

The authors were contacted regarding the concerns raised and provided a replacement Figure 3A, however given the number of duplicated images and the resizing of the images, the Editorial Board stands by the decision to retract. The authors agree to the retraction.

